# Editorial: Umbilical cord milking—benefits and potential harmful effects

**DOI:** 10.3389/fped.2023.1210388

**Published:** 2023-06-07

**Authors:** Hasan Kilicdag, Deniz Anuk Ince, Ayse Ecevit

**Affiliations:** Division of Neonatology, Baskent University Faculty of Medicine, Ankara, Turkey

**Keywords:** umbilical cord management, umbilical cord milking, delayed cord clamping, placental transfusion, hematopoietic stem cells, infants, newborn

**Editorial on the Research Topic** Umbilical cord milking—benefits and potential harmful effects

After delivery, there are several ways to provide a placental transfusion, including umbilical cord milking (UCM), delayed cord clamping (DCC), and delayed cord clamping with resuscitation (DCC-R). Intact umbilical cord milking (I-UCM) is the process of physically expressing umbilical cord blood down a 20–30 cm length of the cord three–four times at a rate of 10 cm per second (1). Cut cord milking (C-UCM) involves clamping and cutting the cord 10–30 cm away from the infant and milking toward the infant (2). With varying degrees of success, UCM has been performed with both cut and intact cords (3, 4). A randomized controlled trial comparing I-UCM with C-UCM and immediate cord clamping (ICC) in neonates born >35 weeks' gestation suggested that I-UCM was a more beneficial choice (5).

However, the lack of information on the effects of UCM on physiology and hemodynamics constitutes a concern (6). With this research topic, we aim to reach more scientific evidence for the benefits and risks of UCM.

Although it has not yet been determined, preterm and term infants with organ damage or deficiencies in hematopoietic cell lines may benefit from stem cell transfusions during UCM (7). The majority of previous studies have excluded newborns with suspected IUGR before birth or infants with prenatal indications of placental insufficiency; as a result, the impact of cord-clamping in these infants is still unclear (8). In order to evaluate the safety, feasibility, and effects of UCM on peripheral blood hematopoietic stem cells, hematological indices, and clinical outcomes in preterm infants with prenatal indications of placental insufficiency, a case-control study was performed by Nagy et al. They discovered that UCM was a safe and advantageous method of umbilical cord management for infants born to mothers who have placental insufficiency. Comparing UCM to historical controls, whose umbilical cords were immediately clamped, showed that UCM increased CD34%, initial hemoglobin (Hb) level, and Hb level at 2 months. Cord milking wasn't linked to a higher incidence of polycythemia or exchange transfusions; however, it was linked to higher peak total serum bilirubin and the requirement for phototherapy.

Umbilical cord blood has been utilized as an alternative source for human transplants since it was shown to contain considerable numbers of hematopoietic stem cells and colony-forming cells (9, 10). The purpose of the study performed by Okulu et al. was to assess the newborns' cerebral oxygenation and delivery room adaptation as well as the concentrations of endothelial progenitor cells and CD34+ hematopoietic stem cells in the placental residual blood volume in healthy term and late preterm infants randomly assigned to undergo DCC, UCM, or ICC. They demonstrated that many lineages of stem cells were lost to the placenta by ICC with a higher residual blood volume, whereas placental transfusion methods such as DCC and UCM provided both higher blood volumes, more stem cell transfers to the infant, and better cerebral oxygenation in the first minutes of life.

The amount of placental transfusion to the newborn is impacted by cord management and spontaneous or assisted ventilation (11). According to a study, cesarean sections (C-section) performed in labor were associated with high newborn hematocrit (Hct), but not those performed electively (12). The study by Kilicdag et al. enrolled women who were admitted for C-section and had a live singleton pregnancy ≥37 weeks of gestation. This study compared various umbilical cord management methods, including physiologic-based cord clamping (PBCC), I-UCM, 30-s DCC, and 60-s DCC, to evaluate their impact on placental transfusion. PBCC was performed immediately after the first spontaneous infant breath. They found that there was no appreciable difference between PBCC, 30-s DCC, and I-UCM in neonates. The neonates' Hb and Hct levels were higher after 60-s DCC than after PBCC.

UCM has been studied as a possible alternative to DCC as it allows for immediate resuscitation after birth. As UCM compared to early cord clamping (ECC) in a cluster-randomized crossover study (MINVI trial), it was suggested to reduce admission to the neonatal intensive care unit (NICU) in non-vigorous newborns delivered between 35 and 42 weeks of gestation. When compared to ECC, UCM did not lead to a decrease in NICU admissions, but it resulted in fewer cases of moderate to severe hypoxic-ischemic encephalopathy, a decrease in the need for therapeutic hypothermia, and higher hemoglobin levels. There was no proof that UCM was harmful as compared to ECC (13). However, there are still concerns about UCM's safety profile, especially for premature babies (14). The review by Koo et al. highlights the known risks and advantages of umbilical cord milking.

Taken together, the evidence presented in this research topic may provide a better approach to umbilical cord management.
